# The Prevalence and Clinical Characteristics of *MYO3A*-Associated Hearing Loss in 15,684 Hearing Loss Patients

**DOI:** 10.3390/genes16010092

**Published:** 2025-01-16

**Authors:** Karuna Maekawa, Shin-ya Nishio, Hiromitsu Miyazaki, Yoko Ohta, Naoki Oishi, Misato Kasai, Ai Yamamoto, Mayuri Okami, Koichiro Wasano, Akihiro Sakai, Shin-ichi Usami

**Affiliations:** 1 Department of Hearing Implant Sciences, Shinshu University School of Medicine, Matsumoto 390-8621, Japan; mkarunakyuudou@me.com (K.M.); nishio@shinshu-u.ac.jp (S.-y.N.); 2 Department of Otolaryngology-Head and Neck Surgery, Tohoku University School of Medicine, Sendai 980-8575, Japan; hmiya1104@yahoo.co.jp; 3 Department of Otorhinolaryngology-Head and Neck Surgery, Tokyo Medical University, Tokyo 160-0023, Japan; yomayomayoko@hotmail.com; 4 Department of Otorhinolaryngology-Head and Neck Surgery, Keio University School of Medicine, Tokyo 160-8582, Japan; oishin@keio.jp; 5 Department of Otorhinolaryngology, Juntendo University, Tokyo 113-8421, Japan; mkasai@juntendo.ac.jp; 6 Department of Otorhinolaryngology, Tokai University School of Medicine, Isehara 259-1193, Japan; ai3sazabon@msn.com (A.Y.); mayuri.okami@gmail.com (M.O.); wasano@tokai.ac.jp (K.W.); 7 Department of Ear Nose and Throat-Head and Neck Surgery, Wakayama Medical University, Wakayama 641-0012, Japan; yhm@sakai-ent.com

**Keywords:** *MYO3A*, non-syndromic hearing loss, progressive hearing loss, late-onset hearing loss, DFNB30

## Abstract

**Background/Objectives:** *MYO3A* belongs to the unconventional myosin superfamily, and the myosin IIIa protein localizes on the tip of the stereocilia of vestibular and cochlear hair cells. Deficiencies in *MYO3A* have been reported to cause the deformation of hair cells into abnormally long stereocilia with an increase in spacing. *MYO3A* is a rare causative gene of autosomal recessive sensorineural hearing loss (DFNB30), with only 13 cases reported to date. In this study, we aimed to elucidate the phenotypes caused by *MYO3A* variations. **Methods:** Massively parallel DNA sequencing was performed on 15,684 Japanese hearing loss patients (mean age 27.5 ± 23.1 years old, 6574 male, 8612 female and 498 patients for whom information was unavailable), identifying nine candidate patients with *MYO3A* variants. **Results:** We identified eight causative *MYO3A* variants by massively parallel DNA sequencing, including six novel variants, and reported nine individuals possessing *MYO3A* gene variants, which is the largest group of non-related patients yet to be detected. Our findings confirmed that *MYO3A* variants cause progressive hearing loss, with its onset varying from birth to the second decade, eventually leading to severe-to-profound hearing loss. **Conclusions:** We clarified that patients with *MYO3A* gene variants present with late-onset, progressive hearing loss. Our findings have enabled us to predict the outcomes of hearing loss in patients with candidate *MYO3A* gene variants and to provide intervention in a timely manner.

## 1. Introduction

Hearing loss (HL) is one of the most common sensory impairments, with congenital cases affecting 1.62 in 1000 newborns [1]. Around 70% of prelingual HL cases have been reported to have a genetic cause, with autosomal recessive sensorineural HL (ARSNHL) being the most common type of genetic HL. Sixty-one genes have been identified to cause this type of HL [2,3]. Children born to consanguineous parents have been found to have a higher incidence of autosomal recessive disorders, including HL. The loci associated with inherited non-syndromic HL are designated as “DFN” (for “DeaFNess”), with the letter “B” indicating autosomal recessive inheritance patterns (DFNB) [4]. The major causative genes of ARSNHL in Japanese patients are *GJB2* (16%), *SLC26A4* (5%) and *CDH23* (4%), and the detection of causative genes for HL has become significantly more achievable through the introduction of massively parallel DNA sequencing (MPS) analysis [5]. However, the elucidation of HL causation becomes more challenging when related to late-onset HL, as multiple factors, such as presbycusis, idiopathic sudden SNHL, environmental risk factors, etc., can be involved. The detection rate of genetic causes in late-onset HL patients is reported to be roughly 20 to 30% [5,6]. Identifying the cause of HL is crucial, particularly if a causative gene is involved, as it allows us to predict its outcome, such as progression, thus enabling timely intervention.

The *MYO3A* gene encodes the myosin Ⅲa protein, which belongs to the unconventional myosin superfamily, and localizes on the tip of the stereocilia of vestibular and cochlear hair cells. Stereocilia are composed of a tightly bundled and crosslinked cytoskeleton actin core and contain mechanosensitive cellular protrusions. The tip region is located at the tip of the stereocilia shaft, containing protein complexes that maintain the link between adjacent stereocilia. The myosin Ⅲa protein is important for structural dynamics, with regular turnover. The maintenance of the length and morphology of stereocilia are crucial to normal hearing, and it is speculated that there is an auto-regulatory mechanism that uses phosphorylation to balance kinase activity and ATPase activity to precisely control myosin Ⅲa concentration at the protrusion tips [7,8,9]. Myosin Ⅲa consists of one extension protein kinase domain, an N-terminal conserved motor domain (head) that contains the ATP and actin-binding regions, a light chain-binding 2 calmodulin-binding IQ neck domain and a C-terminal region tail domain [10]. Several excellent illustrations showing the localization and function of myosin Ⅲa protein in the hair cell stereocilia are available elsewhere [7,8,10,11]. Previous studies have strongly suggested that the myosin Ⅲa protein requires an intact motor and tail domain for tip localization, as well as to induce and elongate actin protrusions [11,12].

The *MYO3A* gene is a rare causative gene of ARNSHL (DFNB30), with only 13 cases reported to date [13]. A *MYO3A* deficit in mice has been reported to cause stereocilia of abnormal length and an increase in spacing between stereocilia rows [7,8,9]. It has been proposed that myosin Ⅲa moves toward the plus end of actin protrusions, docks at the tips, and produces a plus-end directed force that elongates the actin protrusion [14]. Studies in model mice have also shown that loss of function of both *MYO3A* and *MYO3B* leads to profound deafness, whereas the loss of *MYO3A* function alone causes progressive HL similar to DFNB30, the type of HL associated with *MYO3A* variants in humans [15,16]. Although many basic studies have been performed, the detailed clinical characteristics of *MYO3A*-associated HL remain unclear. The clinical phenotypes of *MYO3A*-associated HL varied among cases in previous reports [17,18,19,20,21,22,23,24,25,26], with some cases showing congenital HL, whereas others showed late-onset HL. The severity of HL also varied from mild to profound. Thus, further study is needed to clarify the clinical characteristics of *MYO3A*-associated HL.

In this study, we performed MPS analysis for 15,684 HL patients and identified the patients with biallelic *MYO3A* variants, which is the largest group of patients to be reported to date. In this study, we were able to further our understanding of the clinical characteristics of *MYO3A*-associated HL and evaluated genotype–phenotype correlations through an analysis of the clinical data.

## 2. Materials and Methods

### 2.1. Subjects

In this study, 15,684 Japanese HL patients (mean age 27.5 ± 23.1 years old, 6574 male, 8612 female and 498 patients for whom information was unavailable) were recruited from 102 otolaryngology departments across the country, as detailed in our previous paper [5]. Among these subjects, we selected patients with biallelic *MYO3A* variants through MPS of 158 target genes. Prior to participating in this study, all patients (or from their next of kin, caretaker, or guardian in case of minors or children) provided written informed consent. This study was approved by the Shinshu University Ethical Committee and the ethical committee of each participating institution. Clinical information was obtained from medical charts. Peripheral blood samples were obtained from each individual and from their consenting relatives. This study was conducted according the Declaration of Helsinki. The study protocol was approved by the Ethics Committee of Shinshu University School of Medicine (no.387—4 September 2012, no.576—2 May 2017 and no.718—7 March 2022). The participants were enrolled between September 2012 and November 2024, and MPS analysis was performed between April 2021 and December 2024.

### 2.2. Variant Analysis

Sequencing libraries of the 158 target genes reported to cause nonsyndromic or syndromic HL were prepared with an Ion AmpliSeq Custom Panel (ThermoFisher Scientific, Waltham, MA, USA) using the Ion AmpliSeq Library Kit 2.0 (ThermoFisher Scientific) and the Ion Xpress Barcode Adapter 1–96 Kit (ThermoFisher Scientific), according to the manufacturer’s instructions. The detailed protocol has been described in our previous paper [27]. After the sequencing libraries were prepared, sequencing was performed using the Ion S5 plus system with the Ion 540 Kit-Chef and Ion 540 Chip Kit (ThermoFisher Scientific) according to the manufacturer’s instructions. The sequencing read data were mapped against the human genome sequence (build GRCh37/hg19) using the Torrent Mapping Alignment Program. Following mapping, the DNA variants were picked up using the Torrent Variant Caller plug-in software version 5.16.0.0. After variant calling, their impacts were assessed using ANNOVAR software version 2020-06-08 [28]. The protein-affecting variants (including the missense, nonsense, insertion/deletion and splicing variants) with a minor allele frequency of less than 1% of the 1000 genome database [29], the Genome Aggregation Database [30], the 54,000 Japanese genome variation database (ToMMo 54KJPN) [31], and the 333 in-house controls were selected. All filtering procedures were performed as described in our previous paper [4]. Direct Sanger sequencing was utilized to validate the identified variants. PCR and sequencing primers used in this study were shown in Supplemental Table (Table S1). We also performed copy number analysis based on the read depth data obtained from NGS analysis for all 158 genes as described in our previous paper [32]. The pathogenicity of identified variants was evaluated according to the American College of Medical Genetics (ACMG) standards and guidelines [33] with the ClinGen Hearing Loss Clinical Domain Working Group Expert Specification [34].

### 2.3. Clinical Evaluations

We collected information on onset age, the progressiveness of HL, pedigree and episodes of vertigo from medical charts. Pure-tone audiometry was used to assess hearing thresholds for patients aged 5 years and above, whereas auditory steady state response (ASSR), conditioned orientation response audiometry (COR: one type of the behavioral audiometry), or play audiometry were used for individuals under 5 years old. The pure-tone average (PTA) was determined using the audiometric thresholds at four different frequencies (0.5, 1, 2 and 4 kHz). We divided their HL into four categories: mild (>25 dB and ≤40 dB HL), moderate (>40 dB and ≤70 dB HL), severe (>70 dB and ≤90 dB HL) and profound (>90 dB HL). The type of HL was classified as flat, low-frequency HL, mid-frequency HL, sloping high-frequency HL (gradually lowering 10 dB for high frequency) or precipitous high-frequency HL (higher-frequency thresholds that worsened by at least 20dB per octave) as described elsewhere [35].

## 3. Results

### 3.1. Detected Variations

**Figure 1:**
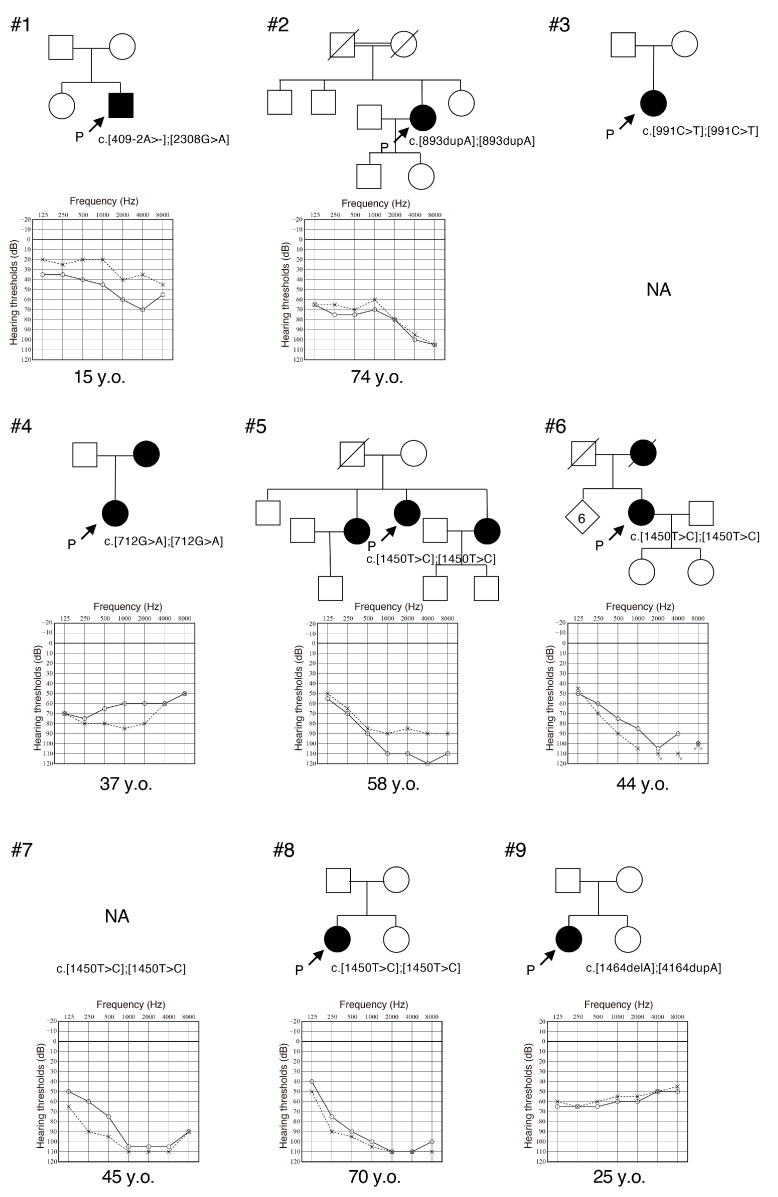
Pedigree and audiograms for the families of each *MYO3A*-associated HL patient identified in this study. The variants identified in this study are indicated in the figure. Pedigrees have been enumerated #1 to #9 for clarification. Solid line: hearing threshold in the right ear; Dashed line: hearing threshold in the left ear.

We identified eight possibly disease-causing *MYO3A* variants, six of which were novel (Table 1). The novel variants consisted of two missense variants, three frame-shift deletion variants and one truncating variant. Two variations were on the kinase domain, three on the motor domain and one was on the tail domain of *MYO3A* (Table 1). The c.1450T>C variant was detected in four patients from three different hospitals (Table 2). There was no apparent consanguinity amongst these four patients, and the identified patients belonged to independent pedigrees (Figure 1).

The allele frequency of all variants identified in this study was less than 0.0007, supporting the threshold defined by the ClinGen HL Clinical Domain Working Group, based on the aforementioned database (Table 1). Based on the ACMG guidelines, four of these novel variants were categorized as likely pathogenic, and two as variants of uncertain significance. As we were not able to conduct segregation analyses on the families of the probands due to a lack of peripheral blood samples, it is possible that the HL was due to other causes, but no other biallelic recessive gene variations were detected by our filtering of the MPS results. The low carrier frequencies in the Japanese control population database (ToMMo 54KJPN) also support the idea that their HL was caused by pathogenic *MYO3A* variants.

### 3.2. Clinical Characteristics of MYO3A-Associated HL

**Figure 2:**
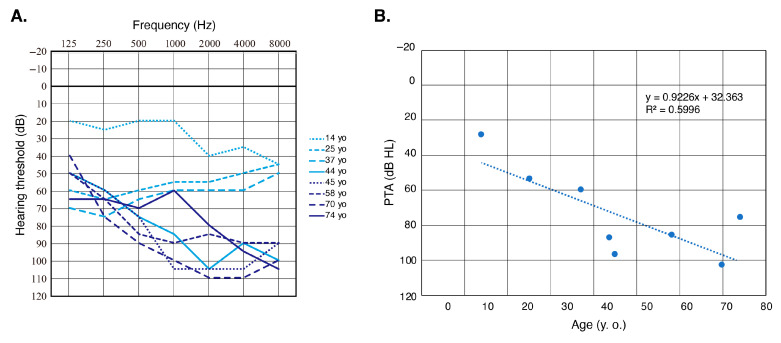
(**A**) Overlapping audiograms from all *MYO3A*-associated HL patients identified in this study. (**B**) Detailed progression analysis of hearing deterioration for patients with *MYO3A*-associated HL. Each dot indicates the pure-tone average (PTA; average of hearing thresholds for 500 Hz, 1000 Hz, 2000 Hz and 4000 Hz) and age of each patient. Dotted line indicates the linear regression.

Nine affected individuals from nine independent pedigrees were detected in this study. *MYO3A* is a relatively rare causative gene, and the prevalence of *MYO3A*-associated HL among Japanese HL patients is 0.06% (9/15,684). We were able to obtain clinical information from eight of these patients, which is summarized in Table 2. The onset age of their HL varied from 10 to 30 years old (mean age: 19.6 years old), and all of them presented with post-lingual deterioration in hearing. The severity of their HL varied from mild to profound, and all patients were aware of HL progression at the time of their genetic testing. Although we were not able to obtain serial audiograms from individual patients, the correlation between age and HL progression was observable on the overlapping audiograms of all individuals (Figure 2A). Assessment of HL progression by scatter plotting the pure-tone average data for our patients clearly indicated the progression of HL with age (Figure 2B). The types of HL were categorized as down-sloping in six, and flat in two patients. Two individuals complained of vertigo, but the specifics are unknown.

## 4. Discussion

**Figure 3:**
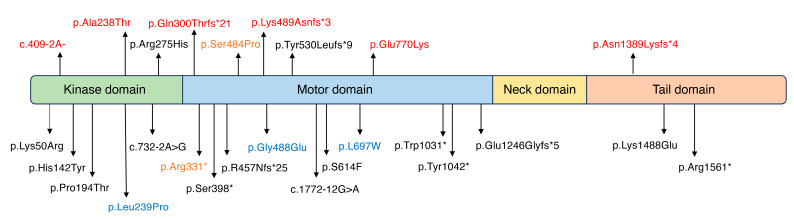
All reported pathogenic *MYO3A* variants and their locations in the *MYO3A* gene. Novel variants identified in this study are indicated in red, variants identified in this study and previous reports are indicated in orange and variants reported to have an AD inheritance pattern are indicated in blue. *: stop codon.

Among the eight possibly pathogenic *MYO3A* variants identified in this study, six were novel. The localization of the identified variants is shown in Figure 3. The position of the variations may influence the genotype–phenotype correlations, but none were observed in this study. For example, it has been reported that damaging effects in the kinase domain cause congenital profound HL [18], yet all patients detected in our study, including those with homozygous variants in the kinase domain, presented with HL onset at over 10 years of age. Further, no effect on phenotypes was observed with the type of amino acid change caused by nucleotide changes.

In this study, we identified nine candidate individuals with HL caused by *MYO3A* variants, which is the largest number of patients to be detected to date. Based on the HGMD professional database [13], there are 41 *MYO3A* variants currently listed as HL causal variants. However, 15 of those variants were not identified as a cause for HL, but were only detected through MPS analysis (i.e., the variant was detected only in the heterozygous state, in vitro fertilization (IVF) screening, IVF donor screening). On the other hand, 13 cases have been reported to have ARNSHL caused by *MYO3A* deficiency (DFNB30), including 15 HL causal variants. We summarized the identified variants and clinical information from previous reports for comparison with the results of this study (Table 3). In our study, all patients presented with late-onset HL and were aware of the progression of their HL, with the overlapping audiograms also showing age-related deterioration in hearing (Figure 2). Unlike the findings in our study, in five of nine reported cases for which information on onset age was available, HL onset was congenital or pre-lingual. The exact ages at which their audiograms were obtained remains unclear, but all patients in the previous reports presented with moderate-to-profound HL (Table 3).

It should be noted that variations in the *MYO3A* gene had been thought to cause ARNSHL (DFNB30), but were reported as a genetic cause of autosomal dominant HL [36,37,38]. To date, most *MYO3A* variants causing ADNSHL have been reported from Brazilian families or from families of South African decent, but this sheds new light on the evaluation of our database, indicating that heterozygous variations might be the cause of HL. However, we face challenges in the verification of this conclusion as segregation analysis is difficult in Japan due to the increase in nuclear families and reduction in the number of births.

In this study, two patients noted their experience of vestibular symptoms. Unfortunately, we could not obtain detailed vestibular testing results for these cases. Due to this limitation of this study, further study is needed, including comprehensive vestibular assessment (caloric testing, cervical vestibular evoked myogenic potential, ocular vestibular evoked myogenic potential and video head impulse testing), to conclude the effects of *MYO3A* gene variants on vestibular function.

It is crucial that we provide adequate information, support and treatment to patients that present with late-onset, progressive HL. Patients will have to adjust their lifestyles, and regardless of the provision of hearing aids and cochlear implantation, deterioration in their quality of life is inevitable. In Japan, patients diagnosed with late-onset or juvenile-onset progressive HL due to pathogenic variations in the *ACTG1*, *CDH23*, *COCH*, *KCNQ4*, *TECTA*, *TMPRSS3*, *WFS1*, *EYA4*, *MYO6*, *MYO15A* and *POU4F3* genes, and who have progressed to severe-to-profound HL, are provided with medical support from the government throughout their life, and we aim to further our research and increase the number of genes for which late-onset progressive HL patient support is applicable.

As future prospects, we believe that the clinical phenotypes of *MYO3A*-associated HL patients clarified in this study will be useful in providing more appropriate clinical management. In particular, most of the cases identified in this study and previous reports showed progressive HL, eventually progressing to severe-to-profound HL. Thus, frequent follow-up and clinical intervention, including the provision of hearing aids and cochlear implantation, should be considered. A further prospective study will be useful in evaluating hearing deterioration through the use of the serial audiometric testing results from the same patient. In addition, the evaluation of the outcomes of hearing aids or cochlear implantation will also be useful.

**Table 3:** Clinical characteristics of *MYO3A*-associated hearing loss patients in previous reports.

Nucleotide Change	AA Change	Inheritance	Onset	Severity	Configuration	Progression	Population	Reference
c.[149A>G];[149A>G]	p.[K50R];[K50R]	AR	Congenital	Profound	NA	NA	Tunisia	[26]
c.[424C>T];[424C>T]	p.[H142Y];[H142Y]	AR	Congenital/ Pre-lingual	Severe-to-profound	NA	NA	South Africa	[23]
c.[580C>A];[1582_1583insT]	p.[P194T];[Y530Lfs*9]	AR	NA	Severe-to-profound	HF_precipitous	NA	Korea	[18]
c.[732-2A>G];[732-2A>G]	p.[IVS8 as A-G -2];[IVS8 as A-G -2]	AR	Second decade	Moderate-to-profound	HF_precipitous	NA	US	[17]
c.[824G>A];[3737_3738delAG]	p.[R275H];[E1246Gfs*5]	AR	NA	Moderate-to-severe	NA	NA	China	[24]
c.[1370_1371delGA];[1370_1371delGA]	p.[R457Nfs*25];[R457Nfs*25]	AR	Pre-lingual	NA	NA	Yes	Palestine	[22]
c.[1450T>C];[3093G>A]	p.[S484P];[W1031X]	AR	6 yo	Severe	HF_gentle	Yes	China	[25]
c.[1777-12G>A];[3126T>G]	p.[IVS17 as G-A -12];[Y1042X]	AR	Second decade	Moderate-to-profound	HF_precipitous	NA	US	[17]
c.[1841C>T];[1841C>T}	p.[S614F];[S614F]	AR	Congenital	Profound	NA	No	China	[20]
c.[3126T>G];[3126T>G]	p.[Y1042X];[Y1042X]	AR	Second decade	Moderate-to-profound	HF_precipitous	NA	US	[17]
c.[4462A>G];[[4681C>T]	p.[K1488E];[R1561X]	AR	Congenital/ Pre-lingual	Profound	NA	NA	Taiwan	[19]
c.716T>C	p.L239P	AD	Pre-lingual	Moderate-to-profound	HF_gentle	Yes	Europe	[38]
c.1463G>A	p.G488E	AD	Post-lingual early-onset	Moderate-to-profound	NA	Yes	African American	[36]
c.2090T>G	p.L697W	AD	10-60	Mild-to-profound	HF_gentle	Yes	Brazil. Portugal	[37]

AA: amino acid, AR: autosomal recessive, AD: autosomal dominant, HF_gentle: sloping high frequency HL, HF_precipitous: precipitous high-frequency HL, *: stop codon.

## 5. Conclusions

In this study, we utilized MPS to identify nine candidate individuals with HL caused by *MYO3A* variations, which is the largest number of patients to be detected to date. *MYO3A* variations cause late-onset HL without any accompanying symptoms. Our findings have confirmed that although onset age may vary from birth to the second decade, the HL will progress, eventually leading to severe-to-profound HL. Although the frequency of *MYO3A*- associated HL is lower than that of other ARNSHL genes, it is essential that we take MPS into consideration for early onset detection to better allow for timely intervention.

## Figures and Tables

**Table 1:** The *MYO3A* variants identified in this study.

Nucleotide Change	AA Change	Exon	Domain	SIFT	PP2	LRT	MutTaster	MutAssessor	REVEL	CADD	ToMMo 54KJPN	gnomAD All	Pathogenicity	Reference
c.409-2A>-		exon6	Kinase	.	.	.	.	.	.	.	1.80E-05	.	Likely pathogenic (PVS1, PM2)	This study
c.712G>A	p.Ala238Thr	exon8	Kinase	T	D	D	D	L	0.451	33	.	.	VUS (PM2, PM3_Supporting)	This study
c.893dup	p.Gln300Thrfs*21	exon10	Motor	.	.	.	.	.	.	.	0.000221	.	Pathogenic (PVS1, PM2_Supporting, PM3_Supporting)	This study
c.991C>T	p.Arg331*	exon11	Motor	.	.	D	A	.	.	.	9.00 × 10^−6^	3.18 × 10^−5^	Pathogenic (PVS1, PM2_Supporting, PM3_Supporting)	[20]
c.1450T>C	p.Ser484Pro	exon15	Motor	D	D	D	D	H	0.905	27.8	4.60 × 10^−5^	3.98 × 10^−6^	Pathogenic (PM3_Strong, PP1_Strong, PM2_Supporting, PP3)	[25]
c.1464del	p.Lys489Asnfs*3	exon15	Motor	.	.	.	.	.	.	.	0.000129	.	Likely pathogenic (PVS1, PM2_Supporting)	This study
c.2308G>A	p.Glu770Lys	exon21	Motor	T	P	D	D	L	0.491	24	1.80 × 10^−5^	.	VUS (PM2)	This study
c.4164dup	p.Asn1389Lysfs*4	exon30	Tail	.	.	.	.	.	.	.	0.000101	.	Likely pathogenic (PVS1, PM2_Supporting)	This study

All variants are indicated on NM_173591. AA: amino acid, PP2: PolyPhen2, MutTaster: mutation taster, MutAssessor: mutation assessor, T (in SIFT): tolerated, D (in SIFT): deleterious, D (in PP2): probably damaging, P (in PP2): possibly damaging, D (in LRT): deleterious, D (in MutTaster): disease causing, A (in MutTaster): disease causing automatic, L (in MutAssessor): low, H (in MutAssessor): high, VUS: variant of uncertain significance, *: stop codon.

**Table 2:** Clinical characteristics of the *MYO3A*-associated hearing loss patients identified in this study.

Family Number	ID	Base Change Allele 1	AA Change Allele 1	Base Change Allele 2	AA Change Allele 2	Inheritance Pattern	Onset	Age	Gender	Severity of HL	Type of HL	Progression	Vestibular Symptoms
1	JHLB-3058	c.409-2A>-		c.2308G>A	p.Glu770Lys	Sporadic	12	15	M	Moderate	HF_gentle	Y	Y
2	JHLB-14312	c.893dupA	p.Gln300Thrfs*21	c.893dupA	p.Gln300Thrfs*21	Sporadic	22	74	F	Severe	HF_gentle	Y	N
3	2402	c.991C>T	p.Arg331*	c.991C>T	p.Arg331*	Unknown	NA	NA	F	NA	NA	NA	NA
4	O-3349	c.712G>A	p.Ala238Thr	c.712G>A	p.Ala238Thr	AD/Mit	13	37	F	Moderate	Flat	Y	Y
5	JHLB-5865	c.1450T>C	p.Ser484Pro	c.1450T>C	p.Ser484Pro	AR	20	58	F	Profound	HF_gentle	Y	N
6	JHLB-12633	c.1450T>C	p.Ser484Pro	c.1450T>C	p.Ser484Pro	AD/Mit	25	44	F	Profound	HF_gentle	Y	N
7	JHLB-13481	c.1450T>C	p.Ser484Pro	c.1450T>C	p.Ser484Pro	Unknown	25	45	F	Profound	HF_gentle	Y	N
8	JHLB-14690	c.1450T>C	p.Ser484Pro	c.1450T>C	p.Ser484Pro	Sporadic	30	70	F	Profound	HF_gentle	Y	N
9	JHLB-889	c.1464delA	p.Lys489Asnfs*3	c.4164dupA	p.Asn1389Lysfs*4	Sporadic	10	25	F	Moderate	Flat	Y	N

AA: amino acid, AR: autosomal recessive, AD: autosomal dominant, M: male, F: female, HF_gentle: sloping high frequency HL, Y: yes, N: no, *: stop codon.

## Data Availability

The datasets used during the current study are available from the corresponding author on reasonable request due to ethical reasons.

## Supplementary Materials

The following supporting information can be downloaded at: https://www.mdpi.com/article/10.3390/genes16010092/s1, Table S1: *MYO3A* primers used in this study.
