# Supplementary material for: The Prevalence and Clinical Characteristics of MYO3A-Associated Hearing Loss in 15,684 Hearing Loss Patients
Source: Genes (Basel). 2025 Jan 16;16(1):92. doi: 10.3390/genes16010092 (PMC11764741; doi:10.3390/genes16010092)
Supplement: Supplementary file 1 [file genes-16-00092-s001.zip › genes-3421271-supplementary.pdf]

Supplemental Table S1. *MYO3A* primers used in this study

exon	forward	reverse
6	aaggagcatgctttgattctg	aaggaaggccattcgaaaaa
8	tgcttgttaaagtcacaaatttca	aagtaaatcgggctgcagat
10	aagcttgggctgagcattta	tgtaagtccccattacctcca
11	ccttcagcctcatcaccttg	ccttctaccaatacccgtgtc
15	gcttaccaaagaagagacatttca	gtttgcttctgcttcctcca
21	cttgaaccttggaggcagag	tcaaatatgcaggctttgtgac
30	tgccaatagaagtaataagctcca	tgtacctctgatagtaactctggatca
